# When screening and diagnosis converge: participant interpretations of additional findings in the 100,000 genomes project

**DOI:** 10.1038/s41431-026-02139-1

**Published:** 2026-05-22

**Authors:** Susie Weller, Anneke Lucassen

**Affiliations:** 1 Centre for Human Genetics, University of Oxford, Oxford, UK; 2 Centre for Personalised Medicine, University of Oxford, Oxford, UK

**Keywords:** Population screening, Medical genomics, Ethics

## Abstract

Genomic science is a central feature of the UK government’s current 10-year health plan for England, which places significant emphasis on prediction and prevention. Although NHS prevention strategies have long been aimed at catching diseases earlier and at a more treatable stage, genomics until now has focussed largely on diagnosis and treatment. At present, there is limited evidence on how patients and publics engage with uncertain and probabilistic genomic information. While the public health benefits of this turn towards genomic screening may take time to establish, insights can be gained from recent large-scale diagnostic initiatives that also offered a screening element. This article draws on a qualitative longitudinal study of 100,000 Genomes Project participants, within which decision-making around the option to receive Additional Findings (AFs) was discussed. By focusing on future health risks in asymptomatic individuals, AFs can be understood as an opportunistic form of screening. Analysis identified three interrelated themes: ambivalence, reflecting anxiety and uncertainty surrounding decisions to receive AFs; inevitability and participant’s own perceptions of patterns of future risk within families; and legacy, capturing motivations framed in collective and relational terms. Participants’ accounts reflected enduring expectations of genomic medicine as a source of certainty and clarity, which sits uneasily alongside probabilistic and uncertain forms of knowledge such screening approaches are likely to produce. Understanding how individuals and families conceive of the potential and pitfalls of employing genomic tests in screening contexts is vital as a range of countries pilot or adopt genomic tools in population health services.

## Introduction

Genomic science is one of the underpinning features of the UK government’s *Fit for the Future:10 Year Health Plan for England* [1], which places significant emphasis on moving away from reactive diagnosis and treatment toward the anticipation of disease and the prediction of future health states. Population health screening is identified as a core mechanism, with plans to establish a Genomics Population Health Service in the next five years. The strategy frames predictive approaches as key to earlier intervention and as a means of alleviating pressure on and transforming the National Health Service [NHS] [1].

Although genomic tests are increasingly promoted for health screening, there is limited evidence on the population-level performance of such predictive tests. The challenges of applying tools that have been evaluated in a diagnostic setting to predict disease in a population remain under-explored [2]. Furthermore, while some studies suggest broad support among patients and healthcare professionals for receiving additional health information [3–5], there is a paucity of evidence on how the range of screening findings and their uncertainties are understood by patients and publics.

As the outcomes of prediction-oriented endeavours, such as Our Future Health, will take time to materialise, it is unlikely we will be able to assess the benefits over harms [6] or explore implications from the perspectives of patients and publics for some years. Valuable insights can, however, be gained from participants’ experiences of recent large-scale genomics initiatives that also employed broad technologies with the aim of diagnosing existing health problems, but at the same time offered findings that might be suggestive of risks of other diseases in the future. These initiatives can help illuminate: (i) how participants respond to the prospect of receiving additional health information; and (ii) how they conceive of the potential benefits and implications.

This article presents a subset of findings from a qualitative longitudinal study (QLR) of individuals and families involved in the 100,000 Genomes Project (100kGP), part of which examined how the prospect of Additional Findings (AFs) were understood within the context of participants’ broader health journeys. Participants were offered AFs from a limited panel of genes associated with ‘actionable’ conditions, intended to support access to preventive health interventions for participants and their relatives [5, 7, 8]. AFs included potential predispositions to various cancers (e.g. Lynch syndrome, breast, ovarian and rare forms), cystic fibrosis and familial hypercholesterolaemia. For children, only AFs associated with childhood-onset conditions were offered.

While we are not suggesting that the offer of AFs is synonymous with population-level screening, there are important parallels, particularly the focus on identifying future health risks in asymptomatic individuals. In this sense, AFs might be regarded as an opportunistic form of screening. Understandings and experiences of AFs are likely to shed light on the issues to which we must attend as prediction becomes increasingly central to healthcare strategy.

## Methods

This article presents a subset of findings relating to AFs from *Journeys through Genomics*; a QLR study exploring the experiences of those affected by the process and outcomes of genomic testing. The study forms part of *Ethical Preparedness in Genomic Medicine* (EPPiGen), an interdisciplinary research programme exploring ethical preparedness within the evolving landscape of genomic medicine [9]. Whilst previous studies have employed retrospective surveys to assess views on AFs across various contexts [10] or focused on the psychosocial impacts of receiving a positive AF [8], our study sought, in part, to understand longitudinally, the social and ethical contexts in which genomic information is received and understood.

### Participants and recruitment

The study focused on the experiences of those offered genome sequencing for a rare disease including rare forms of familial cancer, as well as those connected to and/or supporting someone who had. This article focuses on a purposively selected subsample of 19 cases, all of whom had taken part in the 100kGP. Participants in the wider study were recruited through other routes. The subsample were accessed either via their involvement in a precursor study that focused on issues of consent and confidentiality [11] or through the networks of rare and/or genetic condition patient support and advocacy groups.

### Data generation

Data were generated via a series of semi-structured interviews following participants over time. As the study aimed to longitudinally trace participants’ trajectories, both the number of waves of data generation and the intervals between interviews varied [12] (see Table 1). The first interviews were conducted in 2019 in participants’ homes or community/workplace settings. The onset of social distancing measures during the COVID-19 pandemic, however, necessitated a shift to remote interviewing; a method with which the team was already experienced [13]. Most interviews were conducted via Microsoft Teams, with two participants opting for telephone calls. Remote first interviews were typically longer (mean 85 min) than those conducted in person (mean 59 min). Except for one, all follow-up interviews took place remotely and many participants also provided interim updates by email or engaged in various creative activities. Some of these activities were developed by the team (e.g. the co-production of patient journey illustrations [14] and a collective songwriting workshop), while others were offered by participants unprompted (e.g. sketches of family trees and timelines).

**Table 1:** Overview of the sub-sample of participants and their involvement in the study.

		Involvement in the *Journeys through Genomics* study								
ID	Demographic information	Initial contact	Wave 1 interview	Interim catch up	Wave 2 interview	Interim catch up	Wave 3 interview	Interim catch up	Wave 4 interview	Interim catch up
Aliza	Woman, born 1980s	Jan 2022	Feb 2022	May 2022	–	June 2022	–	–	–	–
Amanda	Woman, age unknown	Jan 2022	Feb 2022	Feb 2022	–	Nov 2024	–	–	–	–
Betty	Woman, age unknown	Nov 2019	Dec 2019	–	Mar 2021	–	–	–	–	–
Charles	Man, born 1950s	Dec 2019	Jan 2020	–	–	–	–	–	–	–
Christina	Woman, age unknown	Jan 2022	Feb 2022	Feb 2022	–	–	–	–	–	–
Claire	Woman, born 1970s	Nov 2019	Dec 2019	Nov 2020	Mar 2021	Nov 2021	–	–	–	–
Clive	Man, born 1950s	Nov 2019	Dec 2019	Feb 2020	Oct 2020	–	–	–	–	–
Hayley	Woman, born 1980s	Jan 2022	Jan 2022	Sept 2024	Oct 2024	Oct 2024	–	Nov 2024	–	–
Jenna	Woman, born 1970s	Mar 2022	May 2022	Oct 2024	–	–	–	–	–	–
Lydia	Woman, born 2000s	Jan 2024	June 2024	Nov 2024	–	–	–	–	–	–
Lynn	Woman, born 1960s	Sept 2021	Sept 2021	Nov 2021	May 2022	June 2022	Jul 2022	Nov 2022	Nov 2022	Nov 2024
Mary	Woman, born 1970s	Jan 2020	Jan 2020	Feb 2020	Nov 2020	June 2023	Jul 2023	Nov 24–May 25	June 2025	–
Michelle	Woman, born 1970s	Jan 2022	Mar 2022	–	–	–	–	–	–	–
Monique	Woman, age unknown	Dec 2020	Dec 2020	June 2022	Nov 2022	Jan 2023	–	Apr 2023	–	–
Rachel	Woman, born 1990s	Jan 2022	Jan 2022	Feb 2022	Mar 2022	June 2022	Nov 2022	Jan–May 2023	–	Nov 2024
Richard	Man, age unknown	Oct 2019	Oct 2019	–	Nov 2020	Nov 2020	–	–	–	–
Shirley	Woman, born 1950s	Nov 2019	Jan 2020	Mar 2021	Apr 2021	–	–	–	–	–
Vivienne	Woman, born 1960s	Jan 2025	Jan 2025	–	Apr 2025	–	–	–	–	–
William	Woman, born 1960s	Sept 2019	Sept 2019	Oct 2019	Oct 2019	–	Nov 2020	Jul 2021	Aug 2021	Nov 2024

An interview guide was used for each wave of data generation. Participants were encouraged to talk about their own journeys through genomics, including their hopes and expectations, encounters with healthcare settings/professionals, involvement of family and how such testing shaped, and was shaped by, the wider context of their lives. Participants were invited to reflect on aspects of earlier interviews, and to think prospectively about their expectations and hopes for the future. AFs were broached during the first interview but were generally discussed in follow up interviews. Key areas included: i) their rationale for opting in/out of receiving AFs; ii) recollections of how AFs had been explained; iii) explorations of what they envisaged AFs included; and iv) discussion of what any outcomes might mean for themselves and/or their family. The longitudinal nature of the study enabled the exploration of change/continuity in recollections and expectations over time.

### Data analysis

**Fig. 1:**
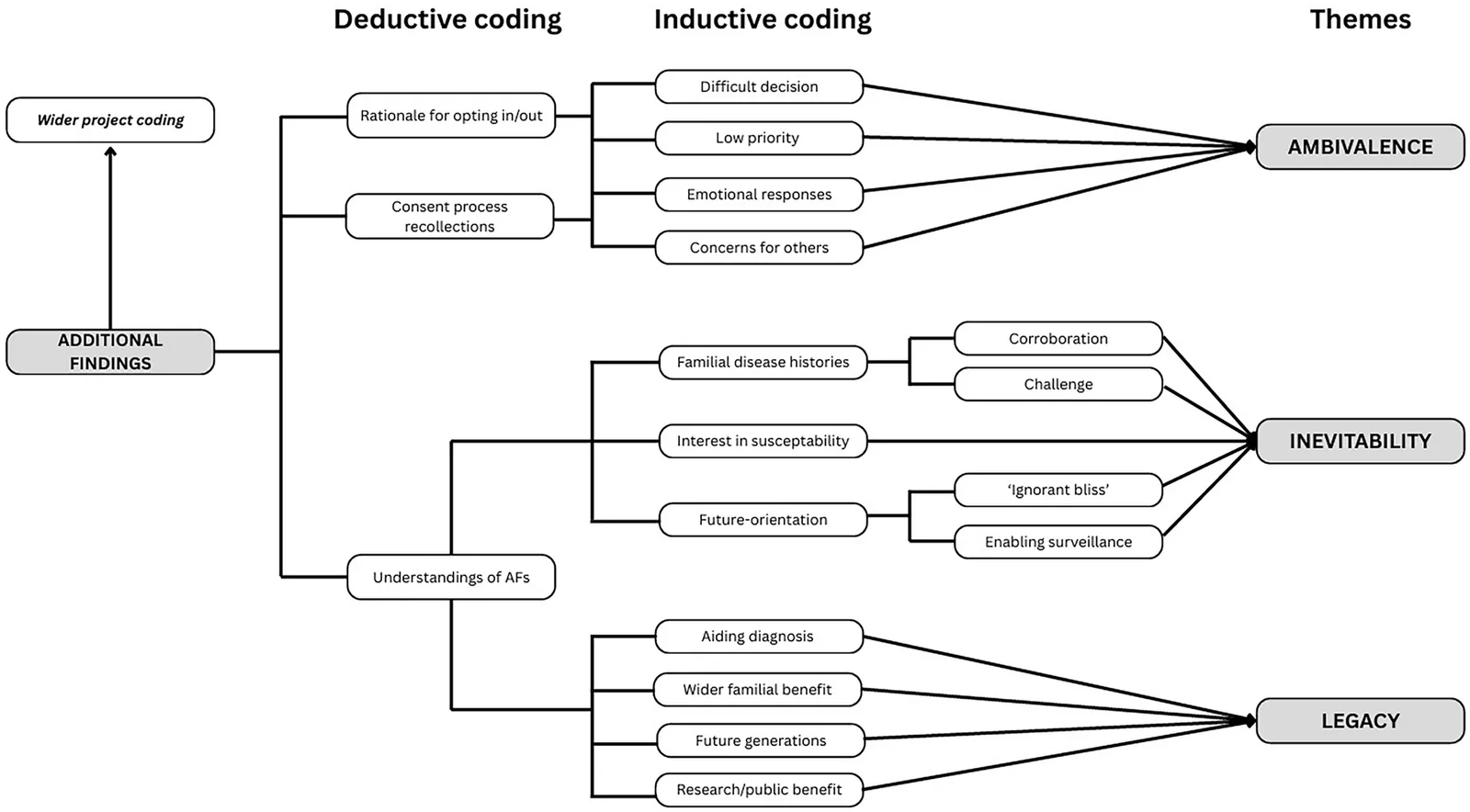
Reflexive thematic analysis. Overview of codes and themes.

Across the wider study a range of analytic approaches were employed including narrative, qualitative longitudinal case and thematic analysis. Reflexive thematic analysis was deemed apt to explore patterns in sense-making relating to AFs, whilst also situating participant’s views within the wider context of their journey through genomic medicine [15, 16]. As part of the process of data familiarisation, multiple readings of the transcripts were undertaken, with attention, for this piece of analysis, paid to the discussion of AFs. Aided by the qualitative analysis software package NVivo (v14), broad brush codes derived deductively from the interview guide prompts on AFs were assigned to help gain a sense of the scope of the material. A more detailed and inductive approach to coding was then undertaken, attending closely to the nuances in participants’ accounts. Through iterative and reflexive engagement with the data, codes were reviewed, refined and clustered around shared patterns of meaning and conceptual coherence. Candidate themes were actively constructed through interpretative engagement with each cluster of codes. Each theme was revisited and refined to ensure analytic coherence and distinctiveness (see Fig. 1 for an overview of the codes and themes). As a result, three interrelated themes were constructed from across participant’s narratives: ambivalence, reflecting anxiety and uncertainty surrounding decisions to receive AFs; inevitability and participant’s own perceptions of patterns of future risk within families; and legacy, capturing motivations framed in collective and relational terms. The inclusion of anonymised interview extracts is intended to be illustrative, grounding the interpretations in the data.

### Ethical considerations

Institutional ethical approval was granted by the Wales Research Ethics Committee (Reference number 21/WA/0344). Our work is guided by an ethic of care with a focus on the situated and evolving nature of research ethics over time [17, 18]. Informed consent was obtained from all participants. Data have been anonymised and pseudonymised.

## Results

The sub-sample comprised 12 index cases and 7 relatives tested to aid the diagnosis of a family member. A total of 38 interviews were conducted, alongside interim correspondence, primarily via email. Whilst most of those involved in our QLR study opted to receive AFs, a small minority decided they simply did not want to know about anything in addition to the medical reason for which testing was being offered. For the most part, this was because they were already grappling with other health issues and/or the health of others. One participant had to withdraw from 100kGP due to issues with their sample. As far as we are aware, none of the participants received news of an AF.

Consistent with previous research [7], during our first wave of interviews participants often struggled to recall what they had agreed to during the consent conversation or indeed whether they had even opted to receive AFs. This is perhaps unsurprising, as others have noted [8], given that AF outcomes were communicated up to seven years after participants had originally enrolled in the 100kGP. All those who elected to receive AFs were informed if a listed genetic variant had been identified or not. Outcomes were disclosed in batches, with members of the same family notified at different times.

For participants in our study, it was not just the passage of time that shaped their recollections but, rather AFs were deemed a low priority compared to the main reason for their involvement. As demonstrated in our previous work, and consistent with findings elsewhere, many participants had already undergone protracted journeys characterised by multiple investigations and tests [19–22]. Akin to the reflections of healthcare professionals responsible for communicating AFs to 100kGP participants [23], participants in our research spoke of surprise at the outcome letter arriving:

‘*I’d heard nothing and it completely came out of the blue. I think it did, I don’t think I had any warning that I was going to receive that, so it was a really positive surprise*’ (Mary, interview 3).

*‘… a few weeks ago, I randomly got a letter in the post … this was just obviously from years ago so completely out of the blue. So you’re sort of looking through the bills and the rubbish that come through the door so it’s… It was a bit of a surprise. And then when I realised it was the 100,000 I was like…(Gasps) There’s like that moment of panic of ‘What’s it going to be, what’s it going to be?*” (Monique, interview 2).

Some participants, such as Hayley (interview 1) had falsely reassured themselves that ‘*no news is good news*’.

### Theme 1: Ambivalence

Even though few could recall many details of what they had agreed to, for some their emotional responses to the decision-making process remained vivid. For example, accounts from parents who had been tested to aid the diagnosis of their child were filled with ambivalence and anxiety about what AFs might mean for others. Monique, who had been on a decade-long quest to understand her son’s symptoms, spoke of the enormity of the decision, eventually choosing to ‘opt in’, not for the potential insights it might offer into her own health risks, but in the hope that it might aid her son’s diagnosis. Monique spoke during her first interview of her ambivalence:

‘*Yeah, because I was nervous about it, and I didn’t know whether to tick it or not. I remember being nervous about it, but then I just thought that I was so desperate to get a diagnosis for <my son> that I thought that, you know, any information would be useful*’ (Monique, interview 1).

By the second interview, Monique had received a letter stating that nothing had been found. She again reflected on the decision-making process and her account was peppered with a persistent feeling of ambivalence:

‘*Do you tick that box, do you not tick that box?” … “If they tell me that I’m predisposed to something do I want to know or do I not want to know?” but then I decided, “Well actually if they say that I’m predisposed to something maybe that bit of information will be useful for looking into <my son’s health*> *… I remember being nervous about it, but then I just thought that I was so desperate to get a diagnosis for <my son> that I thought that, you know, any information would be useful….’* (Monique, interview 2).

On both occasions Monique placed emphasis on her primary motivation for taking part in 100kGP and her quest for an explanation for her son’s symptoms.

Similar anxieties were discussed by others, including Lydia, who was concerned about potential implications for her siblings who were not part of the 100kGP:

‘*But even now I can read back my journal [which] describes I was sort of worrying about things like if I got additional findings. And [if] it turns out I had a variant, like a predisposition to cancer or something like that, and then I was worrying about what that would mean for my sisters and whether they would also get any testing or any kind of monitoring as a result of that result. And so I opted out of additional findings on the basis of a lot of those sort of anxieties*’ (Lydia, interview 1).

Lydia’s concern was that if an AF was identified, whether her siblings, who were not part of the 100kGP, would benefit from screening, and whether they would even want to know about the possibility.

In contrast to dominant policy narratives that position the prospect of knowledge about future health risks as positive, for some participants the decision to take up the opportunity was a challenging one, rife with anxiety and ambivalence. Participants like Monique, who underwent testing to support a child rather than for personal health concerns, found it difficult to view AFs as solely relevant to themselves. Her participation in the 100kGP was motivated by the hope of securing future clarity for her son, with any personal benefit being secondary. This relational framing led to ambivalence, as AFs could not be easily disentangled from the needs of others. Similarly, Lydia questioned whether a positive AF would benefit her siblings and whether they would want to know. Such cases highlight the complexity of decision-making around AFs and the importance of recognising how ‘linked lives’, a concept we have used elsewhere [22], are drawn into the process. When genomic testing for potential future risks is integrated into large-scale initiatives, the primary focus of which is to try to determine a diagnosis, the distinction between diagnostic and predictive ambitions are often lost.

### Theme 2: Inevitability

During interviews, it was common for participants to share their own observations of disease histories within their family, and to surmise about potential hereditary risks to which they felt they might be susceptible. For some, accounts about decision-making in relation to AFs were couched in terms of whether an AF might corroborate or challenge their existing perceptions of familial risks. Clive participated in trio genome sequencing to aid the diagnosis of relatives and to act as a control to rule out other possibilities. Clive’s interest in heritability stemmed largely from his care of/about three generations of his family affected by a heart condition and during the first interview he sketched, unprompted, a family tree outlining incidence of that condition across generations. His main motivation for opting to receive AFs, however, related to his own potential family history. He spoke of wishing to understand his susceptibility to the cancers from which his parents died:

‘*Obviously, I try and be careful about lifestyle and everything, to do what I can in lifestyle so that it doesn’t… not to settle to… But if I’ve got the gene, I’ve got the gene, haven’t I, really, that’s… and if that’s the way I’m going to die, then that’s the way I’m going to die ….’* (Clive, interview 2).

There was a sense of inevitability in his framing of his motivations to receive AFs, particularly those relating to cancer. He felt that it would not necessarily change how he lived his life, but he did allude to the value knowing might have for his brother and his brother’s children.

Some, like Shirley, were convinced of the heritability of several conditions in her family. She identified what she perceived as consistent patterns of cancer and diabetes across both past and present generations and was eager to ensure that all relatives remained vigilant for potential symptoms. This included those in their teens, for whom any risks may not be relevant until later life:

*‘… we’ve got diabetes in the family as well … My grandson was five when he was diagnosed with diabetes …. My uncle’s got it, my gran had it, my mother’s got it, my daughter’s borderline, my son, eldest son, he’s got it…you know, I think it goes through the family*.’ (Shirley, interview 1).

Across the accounts, there was a sense of inevitability, expressed in a variety of ways. From those like Shirley who felt they could see likely patterns of heritable risk to those who believed that if they were going to develop a health condition they would do so anyway and knowing about the possibility would not change anything, except it might have a negative impact on their mental health:

*‘… if something’s going to happen anyway I don’t want to spend the rest of my years until that thing happens worrying about it I’d rather do you know what I mean, I’d rather live in ignorant bliss*’ (Richard, interview 2).

For participants like Clive and Shirley, AFs had possible confirmatory value with the potential to scaffold what they already suspected might have a heritable component. Further information provided by AFs (both positive and negative outcomes) might not necessarily alter the family’s own approach to surveillance. For instance, ensuring relatives, including future generations were informed of the possibility so they could monitor their own health. But, given the commonplace lack of recollection regarding the precise nature of the potential disease predisposing variants included in the 100kGP AFs, there is a danger that a result of ‘nothing has been found’ might give people a false sense of reassurance and reduce their own surveillance practices as individuals and within families.

### Theme 3: Legacy

Whilst participants such as Monique and Lydia expressed a great deal of anxiety about the potential implications of AFs, others saw value in them as a collective resource not just for other family members but also future generations, as we have similarly shown in our work examining understandings of genomic data [24]:

‘*Well again, if it’s something we can do something about, it’s useful to know. Plus, again, it goes back to my kids, so if they did find I have anything, and I am absolutely sure they won’t find anything, but if, again, you just have that gut feeling that if they did, then again, it’s information that might be useful to my children and their children*.’ (Amanda, interview 1).

Claire participated in the 100kGP to try to ascertain a genetic explanation for her diagnosis of colon cancer in her 40s. She had also encountered genetic testing when her son, now in his 20s was born. She opted to receive AFs and when first interviewed Claire focused on the potential personal benefits:

‘*I thought, actually, if you’re going to ask for this stuff, you might as well find out everything there is to know… I mean, I don’t think my life would be massively different as a result. I think I would carry on as I have done throughout, but I think it’s always helpful to be able to tell doctors about these things. If there’s surveillance for anything or there’s something I need to be doing about it, I can*.’ (Claire, interview 1).

But over time her reflections shifted to the wider benefits. Her responses took a more inter-generational view and the potential insights any findings, AFs or otherwise, might offer in terms of understanding the past and her son’s condition, as well as, helping future generations:

‘*I think for me it was always about learning about, understanding the conditions, how they’re caused, and what I could do about it for future generations going forward*’ (Claire, interview 2).

Likewise, for participants such as Betty, Hayley and Vivienne, in addition to an interest in potential benefits for their families, wider societal benefit and the legacy for future generations was apparent in their motivations:

‘*Well, again, for future generations, for research and development*.’ (Betty, interview 2).

‘*We did it for research purpose and curiosity more than anything else*.’ (Hayley, interview 2).

‘*I think it’s a great thing to do and obviously the first reason to do it is for the greater good*.’ (Vivienne, interview 2).

Some saw AFs as part of the wider research endeavour and that opting out might be detrimental to the study:

‘*I didn’t wish to inhibit the project …*’ (Charles, interview 1).

So rather than emphasising whether AFs might provide insights into their own increased risks of developing a health condition, many seemed to prioritise the potential benefits for others, and the endeavour as a legacy for future generations.

100kGP participants were offered AFs on the basis that any outcomes might enable them to access health interventions, but as these examples serve to illustrate, it was more commonplace for participants to articulate their motivations in more collective and relational terms within but also beyond their families. In doing so, visions of genomic testing as providing definitive answers or certainty were evoked. As we have shown in related work, individuals encounter genomic testing with pre-existing ideas about genomics, often formed over time and from multiple sources, which is likely to shape how the possibilities are envisaged [25]. Some of the examples here suggest that prior healthcare encounters also shape ideas about what is valuable and what is possible.

## Discussion

Across many national contexts widespread political and economic faith is being placed in the transformative potential of genomics for healthcare, with recent policy increasingly oriented toward the use of genome sequencing for prediction and population-level screening [1]. Within the UK, considerable investment has already been made in large-scale screening-oriented programmes, including the Generation Study [26] and Our Future Health [27]. Yet the implications of these initiatives for individuals and families are unlikely to be fully understood for many years. In the meantime, there is value in examining qualitative accounts from those involved in other recent national genomic initiatives.

The 100kGP provides a salient example. Although primarily focused on the diagnosis of rare diseases and cancers, it also offered participants the option to receive additional information indicative of future health risks. This gives valuable insights into if, and how, people conceive of differences in diagnosis and population-level screening, when it comes to genomics. Participants were asked to consider whether they wished to receive such AFs, requiring engagement with uncertain and prospective forms of knowledge. Our analysis of QLR data highlights two key areas to which population-level genomic endeavours ought to be attentive.

The first concerns participants’ enduring expectations of genomic medicine as a source of clarity and certainty. Even those who, after several years, had yet to receive an outcome for the original reason for testing continued to hold views shaped by deterministic narratives of genomics. Many participants expressed ambivalence about opting for AFs, simultaneously overestimating and feeling anxious or optimistic about the predictive power of any potential outcomes. These expectations of certainty generated concern that something definitive might be identified with implications for themselves or others, despite the likelihood of receiving a positive AF being very low. This concurs with Halverson’s (2024) argument that “As exome- and genome-scale tests begin to replace smaller panels, and as interpretation driven by clinical indication gives way to the return of ‘incidental’ findings  (...) patients will face even greater chances of receiving uncertain results” [28]. As policy increasingly shifts towards prediction, these findings suggest that entrenched expectations of certainty sit uneasily alongside the probabilistic and uncertain forms of knowledge such approaches are likely to produce.

The second concern relates to expectations about the scope of future health risks that can be revealed through genomic testing. Misconceptions about what was being offered, combined with the length of time between consent conversations and the receipt of outcomes, meant that many participants did not recall or fully understand what information might be communicated [7, 8]. The absence of AFs was sometimes interpreted as a definitive reassurance of health, with potential implications for how individuals and families approached health surveillance. Pre-existing familial narratives about illness and inheritance strongly shaped expectations, with the danger that a lack of clarity about the scope might provide false reassurance. As our analysis has demonstrated, some of those who elected to receive AFs were motivated by a sense of solidarity, anticipating potential benefits for relatives and wider publics, echoing work on solidarity and collective obligations in genomic medicine [29]. As population-level genomic screening expands, it will be essential to avoid overpromising and to clearly communicate not only the scope of these tests but also the current lack of evidence supporting the utility of such tools and approaches in screening contexts [2].

In conclusion, as population-level genomic approaches accelerate, differences between screening and diagnoses risk being missed and the temptation to treat genomic AFs as a definitive diagnosis rather than tentative signals will only grow. There is surprisingly little evidence that genomic tools improve outcomes in screening contexts, making it all the more important to understand how individuals and families interpret the promises and pitfalls of their use. If population health services are to integrate these tools responsibly and equitably, we must attend closely to how individuals and families make sense of these developments.

## Data Availability

Due to the sensitive nature of the interviews, the dataset on which this article draws, is not publicly available. Data is available upon reasonable request, and subject to ethical review and compliance with participant consent.
